# On the Coverage of Bus-Based Mobile Sensing

**DOI:** 10.3390/s18061976

**Published:** 2018-06-20

**Authors:** Pedro Henrique Cruz Caminha, Rodrigo de Souza Couto, Luís Henrique Maciel Kosmalski Costa, Anne Fladenmuller, Marcelo Dias de Amorim

**Affiliations:** 1 Poli/COPPE—Universidade Federal do Rio de Janeiro, Av. Athos da Silveira Ramos 149, Rio de Janeiro 21941-972, Brazil; cruz@gta.ufrj.br; 2 DETEL/PEL—Universidade do Estado do Rio de Janeiro, Rua São Francisco Xavier 524, Rio de Janeiro 20550-013, Brazil; rodrigo.couto@uerj.br; 3 LIP6/CNRS—Sorbonne Université, 4 Place Jussieu, 75252 Paris, France; anne.fladenmuller@lip6.fr (A.F.); marcelo.amorim@lip6.fr (M.D.d.A.)

**Keywords:** wireless sensor networks, coverage, vehicle-based sensing

## Abstract

A cost-effective approach to gather information in a smart city is to embed sensors in vehicles such as buses. To understand the limitations and opportunities of this model, it is fundamental to investigate the spatial coverage of such a network, especially in the case where only a subset of the buses have a sensing device embedded. In this paper, we propose a model to select the right subset of buses that maximizes the coverage of the city. We evaluate the model in a real scenario based on a large-scale dataset of more than 5700 buses in the city of Rio de Janeiro, Brazil. Among other findings, we observe that the fleet of buses covers approximately 5655 km of streets (approximately 47% of the streets) and show that it is possible to cover 94% of the same streets if only 18% of buses have sensing capabilities embedded.

## 1. Introduction

Gathering data is an important aspect of urban computing and a key factor to build smart cities [1]. The Internet of Things (IoT) paradigm, an important tool for smart city sensing, enhances daily life objects with communication, processing, sensing and actuation capabilities [2]. Nevertheless, achieving city-wide coverage with static sensors might lead to prohibitive costs, given the necessary number of sensors. Additionally, gateways in charge of collecting data from sensors must also cover the whole city, leading to an also inflated number of gateways.

A promising solution is to help cover the city with mobile sensors. This strategy allows each sensor, individually, to cover a wider area and benefit from the fact that sensor readings can be intermittent in the majority of the cases [3]. Furthermore, sensor nodes can perform opportunistic communication with gateways, reducing the amount of necessary gateways [4]. Finally, by leveraging existing mobility, such as that of buses, mobility is acquired with negligible costs [5].

One aspect that we must be careful about is that applications atop a bus-based mobile wireless sensor network may have specific requirements in terms of data completeness, timeliness [6] and data granularity [1]. In the case of spatial coverage, completeness and granularity are related to the knowledge of the complete area of the city and to what extent the measurements are spread through the city. Furthermore, acquiring measurements about different areas can reduce noise and uncertainties about neighboring regions [7].

In this paper, we model the spatial coverage of a bus-based mobile wireless sensor network based on a large-scale, real-world mobility dataset in the megalopolis of Rio de Janeiro, Brazil. We propose a coverage model that relies on the fact that buses have fixed trajectories. We also propose a mixed-integer linear programming (MILP) formulation to determine the minimum subset of buses that must have sensing nodes embedded while keeping the same level of coverage as the complete fleet.

As a summary, our contributions are:

- We propose a model of the spatial coverage of a city-wide bus-based sensor network.
- We solve an optimization problem to maximize spatial coverage when the number of buses participating in the system is limited.
- We evaluate and validate the model using a large-scale dataset from a real scenario.

This paper is organized as follows. We propose our model in Section 2 and describe the case study we consider in Section 3. In Section 4, we apply our optimization model to the case study and analyze the results. We postpone the related work to Section 5 in order for the reader to gather enough background and better understand the originality of our contribution. Finally, we conclude the paper in Section 6 and point out future research directions.

## 2. Coverage Model

This work falls in the context of the SensingBus initiative, which takes advantage of preexisting transport infrastructure, with minimal overhead, to add mobility to the sensors [5]. In SensingBus, buses collect data and use the Vehicle-to-Infrastructure (V2I) paradigm to deliver the gathered data in an opportunistic fashion [8]. SensingBus is built on a three-level architecture. At the first level, buses with embedded sensors gather data, following the IoT paradigm. Different environmental measurements are gathered and temporarily stored together with their coordinates and measurement time. At the second level, gathering points located at bus stops receive data from incoming buses and send these data to the third level, using the Internet. The second level follows the fog computing paradigm, pre-processing data before they are sent through the Internet. At the third level, a cloud service receives data from all the bus stops, processes the data and serves them to the final users. Users can access data using either an API or visualizing data plotted on a map.

Buses follow predictable paths that, in general, do not reach every single street of a city. In this sense, it is important to model the spatial coverage of buses in order to predict the coverage of a bus-based mobile sensor network. For the sake of readability, the notation we use in this paper is summarized in Table 1.

We divide each street into non-overlapping parts that we call street sections. A street section must be a part of a street with a single entrance, a single exit and a single possible path inside it. An example of a street section is a portion of street between two consecutive corners. A similar definition can be found in the work by Ali and Dyo [9].

We denote by I the set of all the street sections in the city and by J the set of buses. The path of a given bus is composed of street sections. If the path of a bus j∈J contains a street section i∈I, the section *i* can be covered by the bus *j*. The sections covered by bus *j* compose the set $T_{j}$. Although different buses may follow different paths, a path of a bus might overlap with the path of other buses. Therefore, a single street section *i* might be covered by more than one bus. We denote by $N_{i}$ the set of buses that cover the street section *i*.

It is also possible that some street sections remain uncovered because no buses cross them. We suppose that these street sections are covered by a complementary sensing system and are then out of the scope of our model.

### 2.1. Coverage as a Function of Street Sections

**Figure 1:**
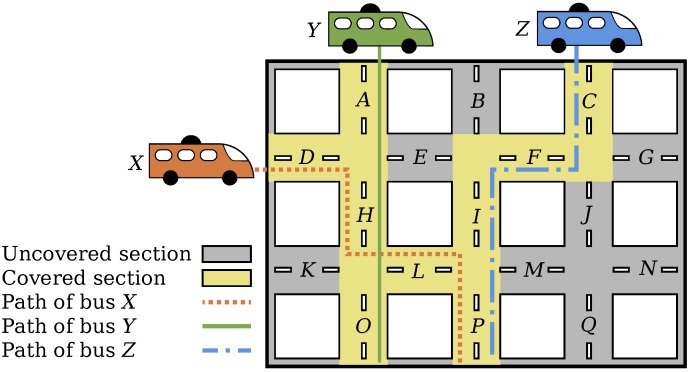
Coverage of street sections by buses equipped with sensors.

Our objective is to determine the subset of buses that, if equipped with sensing devices, denote this subset $J_{s}\in J$. We can now define the set of covered street sections $I_{s}$ as:

(1) $$I_{s}=\underset{j\in J_{s}}{⋃}T_{j},$$

where J is the set of buses that carry a sensing device and $T_{j}$ is the set of street sections covered by bus *j*. Figure 1 illustrates this situation. In this figure, the path of bus *X* is the set of street sections $T_{X}=\left\{D,\;H,\;L,\;P\right\}$; the path of bus *Y* is the set $T_{Y}=\left\{A,\;H,\;O\right\}$; and the path of bus *Z* is the set $T_{Z}=\left\{C,\;F,\;I,\;P\right\}$. Therefore, the set $I_{s}$ of covered street sections is the union $T_{X}\cup T_{Y}\cup T_{Z}=\left\{A,\;C,\;D,\;F,\;H,\;I,\;L,\;O,\;P\right\}$.

Street sections might have different lengths. For this reason, coverage *C* should also integrate the length of every section:

(2) $$C=\sum_{i\in I_{s}}a_{i},$$

where $a_{i}$ is the length of section *i*.

For modeling purposes, it is possible to associate a variable $y_{i}$ with every street section i∈I, such as:

(3) $$y_{i}=\left\{\begin{array}{cc} 1, & \mathrm{if}\;i\in I_{s},\\ 0, & \mathrm{otherwise}. \end{array}\right.$$

We can refine then Equation (2) such as:

(4) $$C=\sum_{i\in I}a_{i}y_{i}.$$

If all buses in the city have embedded sensing devices, i.e., $J_{s}=J$, we obtain the maximum coverage. Nevertheless, this situation may generate prohibitive installation and maintenance costs. Additionally, an area that is already covered might be visited several times, wasting network resources. In this sense, for a given budget, we need to limit the number of buses equipped with sensing nodes. Hence, the problem is to choose which buses should be equipped with the available sensing nodes.

Let J be the set of buses and *p* be the number of sensing nodes, defined by the available budget. We need to select a subset of buses $J_{s}\subset J$ such that $|J_{s}|=p$. Since buses may cover different sets of street sections, with different lengths, the choice of $J_{s}$ may affect the coverage of the network. Let also $N_{i}$ be the set of buses that cover *i* if equipped with sensing capabilities. Thus, section *i* is covered by bus *j* if $j\in N_{i}$ and $j\in J_{s}$. The problem, described next, is to select $J_{s}\subset J$, with cardinality *p*, to maximize the coverage of Equation (2).

### 2.2. Mixed Integer Linear Programming Formulation

We model the problem described before as a Mixed Integer Linear Programming (MILP) problem. The problem is formulated as an MILP because of its complexity and because it is possible to either solve the problem optimally or, for too large instances, estimate the gap between a suboptimal solution and the best possible solution. The MILP formulation is represented as follows:

(5) $$\begin{array}{cc} \mathrm{maximize}:\; & \sum_{i\in I}a_{i}y_{i} \end{array}$$

(6) $$\begin{array}{cc} \mathrm{subject}\;\mathrm{to}: & \sum_{j\in N_{i}}x_{j}\geq y_{i},\;\forall i\in I; \end{array}$$

(7) $$\begin{array}{cc} & \sum_{j\in J}x_{j}=p; \end{array}$$

(8) $$\begin{array}{cc} & x_{j}=\left(0,1\right),\;\forall j\in J; \end{array}$$

(9) $$\begin{array}{cc} & y_{i}=\left(0,1\right),\;\forall i\in I. \end{array}$$

The objective function of Equation (5) maximizes the spatial coverage defined in Equation (4). Equation (6) states that a street section *i* is covered (i.e., $y_{i}=1$) if the system selects at least one of the buses that pass through *i*. Since this is a maximization problem, there is no need to limit a minimum value for variables $y_{i}$, because these variables contribute positively to the objective function, assuming the maximum possible value. Equation (7) ensures that the number of chosen buses is *p*. Equations (8) and (9) define $x_{j}$ and $y_{i}$ as binary variables. When the problem is solved, a variable $x_{j}=1$ indicates that bus *j* is chosen to receive a sensing node, and $x_{j}=0$ indicates that bus *j* should not carry a sensing node; respectively, $y_{i}=1$ indicates that street section *i* is covered by the current configuration, while $y_{i}=0$ indicates that the street section *i* is not covered.

### 2.3. Maximal Covering Location Problem

We show that the problem of choosing a limited set of buses while maximizing coverage is equivalent to the maximal covering location problem [10]. In MCLP, there is a set I of demands distributed in space. For every demand i∈I, there is a value $a_{i}$ related to the benefit of satisfying demand *i*. Demands must be satisfied by a set J of installation candidates, also distributed in space. The MCLP defines that an installation *j* can satisfy a demand *i* if and only if the distance between *i* and *j* is less than or equal to a given distance *S*.

Given this distance requirement, it is possible to determine a set $N_{i}\subset J$ of candidates that satisfy demand *i*. Additionally, there is a limit on the number of installations that can be built, denoted by *p*. The objective of MCLP is to choose a subset $J_{s}\in J$ to build installations, maximizing the benefit of the satisfied demands, such that $|J_{s}|$ equals *p*.

The transformation of our problem into an MCLP consists of considering street sections as demands and buses as installation candidates. The length of the street section *i* is considered as the benefit $a_{i}$ of satisfying demand *i*. The inverse transformation is also possible by transforming demands into street sections, installation candidates into buses and the benefit $a_{i}$ of satisfying demand *i* into the length of street section *i*.

The bidirectional transformation proves the equivalence of both problems. The MCLP is an NP-hard problem [10]. Since there is a bidirectional transformation between the studied problem and the MCLP, our problem is also NP-Hard.

## 3. Case Study

We assess the behavior of our model by analyzing the street coverage in the city of Rio de Janeiro, Brazil. Our analyses rely on real data containing the positions of all buses in the city for a day-long period. In addition, we use these data to build the input for the MILP formulated in Section 2.2, which consists of:

- Set J, containing all the buses in the city;
- Set I, containing all the street sections in the city;
- Set $N_{i}$, for every i∈I, containing all the buses that can cover section *i*;
- Parameter $a_{i}$, associated with the lengths of every i∈I.

### 3.1. Data Gathering

The government of Rio de Janeiro offers an open API (Application Programming Interface) that delivers GPS data of the buses in the city [11]. The basic information is a tuple containing the bus identification, its GPS coordinates and the timestamp of the acquisition of this information. Other elements are also available, such as the bus line identification and the bus instantaneous speed, but we do not consider them in this work.

**Figure 2:**
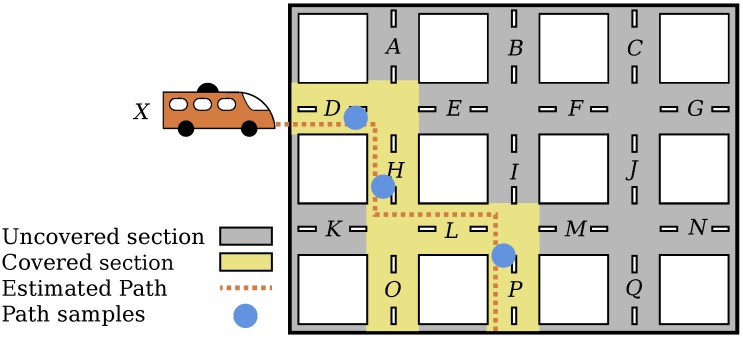
Reconstruction of a bus path using samples as input for Google Snap to Roads.

In this paper, we assume that buses follow fixed paths and that departure intervals might vary in the course of a day, but the same schedule is followed in all weekdays. We have built a script to gather data from the API during 24 h, between 0:00 h of 10 May 2017 and 0:00 of 11 May 2017, a Wednesday. The coordinates of each bus are ordered in time, resulting in a sampling of their paths, every minute. Figure 2 illustrates the sampling of the path. We refer to this dataset as the gathered dataset.

### 3.2. Data Pre-Processing

The gathered dataset needs to be pre-processed before serving as an input for the formulated MILP. The pre-processing consists of evaluating the path of each bus (i.e., a set of street sections) and in estimating the length of each street section. To perform these steps, we use the API Google Snap to Roads [12]. This API receives a list of ordered coordinates as the input, which is a sampling of the path traveled by a vehicle. It returns the most likely path followed by a vehicle according to the input coordinates, in the form of new coordinates, adjusted to the topology of the roads. The work by Ali and Dyo uses a similar approach to obtain the set of streets that can be covered by each bus route [9]. The difference between both methods is explained in Section 5.

Google Snap to Roads also associates every new coordinate to a place id, which represents a road segment. Road segments are data structures used by Google to define segments for their Road API [13]. The Road API is used to give drivers indications about routes and directions. Therefore, a road segment is a street section of our model, since every point on a street is mapped into one, and just one, road segment. Figure 2 illustrates the processes through which samples of the path followed by bus *X* are transformed into a path and associated with street sections (D,H,L,P).

To accomplish the pre-processing goals, three procedures are employed: First, we filter the gathered positions that are identified as noise; second, we use the API Google Snap to Roads to obtain a corrected estimation of bus paths, as coordinates associated with road segments; third, we estimate the length of the road segments obtained in the second procedure.

It is important to underline that the second procedure may require a significant amount of time before completion, as it is proportional to the number of data points in the bus paths. In addition, GPS coordinates gathered from the buses are error-prone [14]. Therefore, the first procedure aims to eliminate positions that are similar to noise or that are unnecessary. This is possible since we have detected data points indicating that some buses were parked at bus garages: those generate data points even when they were not in service. Using this filter, the positions of the same bus path are ordered, and if the distance between two points is smaller than a certain threshold, we can filter the second position out. The first position is never removed, and subsequent positions are compared only to points that are already inside the resulting dataset. We pick 10 m as the threshold because it is a commonplace error range of commercial GPS devices [14]. After this filtering phase, the buses have some positions removed from their paths, resulting in fewer positions to the next procedure. In case a bus has all but one position removed from its path, the bus is considered as stopped and removed from the dataset.

In the course of a single minute, a bus can travel more than one street section. With this regard, simply mapping the gathered positions of a bus to the corresponding section may not reflect the complete coverage of this bus. The second step uses the API Google Snap to Roads and transforms each filtered path, obtained in the previous procedure, into a sequence of coordinates adjusted to the street topology. Google Snap to Roads also associates every adjusted position to a road segment. In the case that an input coordinate does not fall within a street segment, Google Snap to Roads approximates the input coordinates to the nearest street segment. Therefore, the only way GPS errors can affect the path estimation is if the input coordinates are closer to some street segment that is not the previous or the next in the path. Since GPS errors are about 10 m [14], it is reasonable to expect that the coordinates are precise enough for Google Snap to Roads to discover the street segment where the bus was originally traveling when its position was sampled. We refer to an adjusted coordinate associated with a road segment as an adjusted position. The dataset obtained after the pre-processing is called the estimated dataset. Section 3.3 performs an analysis of the gathered and estimated datasets.

### 3.3. Data Analysis

The objectives of gathering and pre-processing real data from buses are to use it as the input to the problem formulated in Section 2.2. To understand the considered scenario, it is important to perform an analysis of the data in terms of its size, before and after the preprocessing. Table 2 exhibits some attributes of the gathered and estimated datasets.

The gathered data contains 5,496,878 positions, obtained from 6075 buses. The filter removes 1,384,925 positions and 328 buses (i.e., about 25% of positions and 5% of buses are removed); thus, we finally dispose of 4,111,953 positions and 5747 buses. After these positions are applied to Google Snap to Roads, we obtain 52,250,671 estimated positions. The increase in the number of positions occurs because Google Snap to Roads estimates a smooth path for the vehicle. As a consequence, it may generate several positions between a pair of input coordinates. The estimated positions are distributed in 95,992 street sections. The sum of the estimated lengths of all street sections is 5655 km. According to the government of Rio de Janeiro, the city has a total of 10,577 km of streets. Hence, it is possible to estimate that the total fleet of buses is capable of covering 53% of the streets.

**Figure 3:**
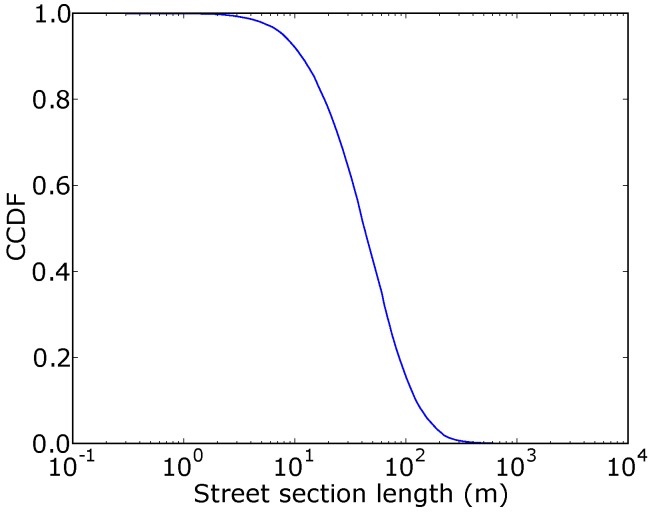
Distribution of estimated street section lengths captured with Snap to Roads.

In this paper, the coverage obtained with a given number of buses is compared to the coverage of all the buses, using a relative coverage. We define the relative coverage as the coverage obtained divided by the coverage when all buses are equipped with sensing nodes, expressed in percentages. In this way, it is possible to better evaluate the effect of the different budgets, in terms of sensing nodes. Figure 3 illustrates the CCDF (Complementary Cumulative Distribution Function) of the estimated street section length. We note that the majority of lengths lie between a few tens to a few thousands of meters. The street sections with the biggest lengths were checked manually, for consistency. The three biggest street sections range from 1780 to 2146 m. Those correspond to freeways that cross the city.

The Union of Bus Companies of Rio de Janeiro (i.e., Rioônibus) also makes available an estimate of the distance traveled by the fleet over the year [15]. Using the most recent records (January–August 2016), it is possible to evaluate the obtained length estimations. According to the data, a bus travels an average of 218 km per day. In our estimation, the average bus travels 175 km on a day. Since we are performing a coverage analysis, it is safe to conclude that we make a pessimistic estimation.

### 3.4. Experiment Execution

After data are gathered, pre-processed and validated, they are used as the input to the problem. The problem is solved using IBM CPLEX 12.5.1. This tool works finding solution candidates and upper limits to the solution, making it possible to estimate the maximum gap between the best solution found and the best solution possible. Given the complexity of the problem, we have configured the solver to return the best solution found when the gap between the upper limit and the best solution found is within 2.0% of the upper limit. The gap of 2.0% was chosen because it was the smallest gap obtained for every tested value. This gap was obtained considering the best equipment available, an Intel Xeon E5-2650 with 264 GB of RAM. The values used for the budget for the number of buses to be equipped with sensing nodes (in the model, denoted by *p*) are 2, 4, 8, 16, 32, 64, 128, 256, 512, 1024, 2048 and 4096. The total number of buses is 5747.

## 4. Results

**Figure 4:**
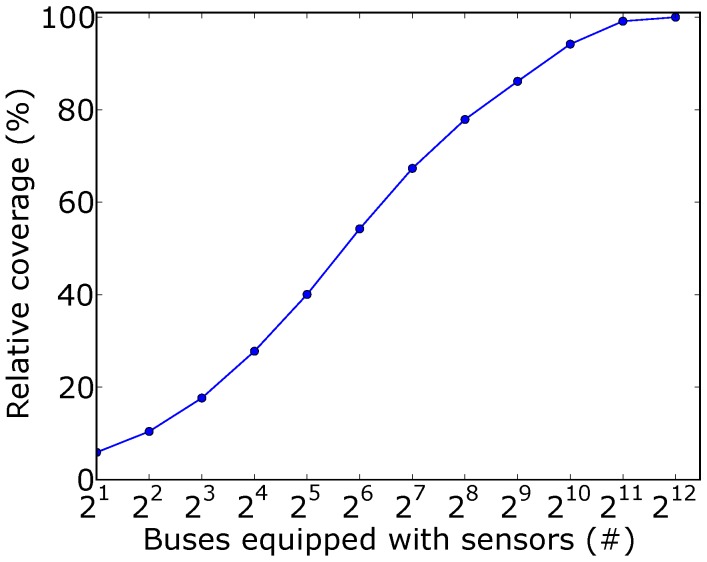
Relative coverage of the buses equipped with sensors.

We show in Figure 4 the relative coverage for different budgets of sensing nodes. As explained in Section 3.3, the relative coverage was calculated with respect to the coverage when all buses are equipped with sensing nodes. The horizontal axis is in logarithmic scale.

We observe from the plot that 1024 buses, or approximately 18% of the bus fleet, were able to cover at least 94% of the streets served by buses. This is equivalent to 5060 km of streets. It is also worth noting that with 32 buses, we were able to cover about 40% of the total covered area. Hence, it is possible to use such a small subset of the buses to build a prototype of the complete service and yet cover 40% of the target area. This somehow shows that it is possible to adopt an incremental deployment of such a sensing system.

The focus of the optimization problem was on maximizing the spatial coverage of the network. Nevertheless, mobile sensing poses a trade-off between spatial and time coverage [3]. Even though the area reached by each sensor grew, some areas were not sensed the whole time. To evaluate the time coverage, we count the number of times the same street section was visited by any bus equipped with a sensing node. The number of visits to a given street section influences the likelihood of detecting some event of interest in this section or determines the freshness of sensed data. Additionally, a larger number of visits also improves the efficiency of algorithms for noise reduction and value prediction [7,16].

**Figure 5:**
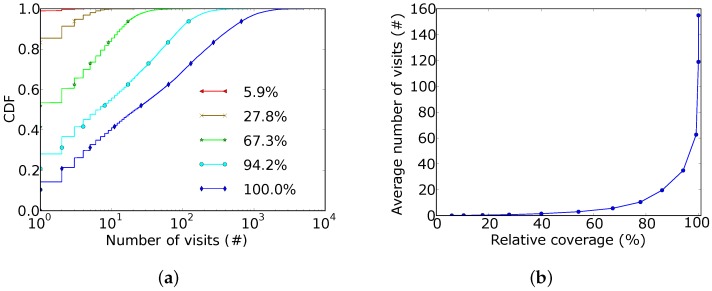
Sensing frequency of street sections throughout a day. (**a**) CDF of the amount of times the same street section was visited, for different coverage proportions; (**b**) average of times a street section was visited, as a function of the coverage proportion.

To estimate the effects of the proposed optimization on the time coverage, we evaluated the number of times each street segment was visited in our dataset, for different values of the relative coverage. Figure 5a shows the CDF of the number of visits of the same street segment for the relative coverages of 5.9%, 27.8%, 67.3%, 94.2% and 100%. These relative coverages correspond to the use of 2, 16, 128, 1024 and 5747 buses, respectively. The horizontal axis is in logarithmic scale. It is possible to observe that when the relative coverage was 5.9%, the covered street sections obtained at most three visits, but when the relative coverage was 94.2%, more than 70% of the covered street sections were visited more than once.

Another way to visualize the information on time coverage is represented in Figure 5b. This figure shows the average number of visits received by each street section in the course of a day as a function of all the relative coverages obtained in our experiments. We observe that the growth rate increased when relative coverage was around 50%. It is also worth noting that, after 50% of relative coverage, the average number of visits grew more rapidly than the spatial coverage. This result reinforces the idea that it is possible to incrementally deploy the sensing system. First, minimal service is established and few applications are supported, since some applications might need a greater number of visits per day; later, more equipment is integrated into the system, enabling new applications and spreading the service to more areas of the city. In a companion paper, we proposed an algorithm to mitigate the network costs of such a system, choosing a limited number of bus stops to place gateways while minimizing the delivery delay caused by opportunistic communication [17].

We would like to refer to Zanella et al. who advocate that a sensing frequency of two measurements per hour is enough for air quality monitoring application [1]. Using this requirement and the expected sensing intervals for the system, it is possible to conclude that equipping 1024 buses with sensing nodes is enough to support applications such as air quality or traffic control to about 40% of the covered streets.

## 5. Related Work

Mobile sensing using urban vehicles is a hot topic in the literature. The BusNet project employs urban buses to measure environmental conditions of streets and roads where vehicles travel [18]. Buses are used as sensing nodes, but also as data mules. Gateways placed at regional bus stops accumulate data and deliver data to buses heading to the main bus stops. The project does not consider the coverage of the system. A prototype was built to evaluate road conditions.

There are initiatives for data gathering with urban vehicles in a broader sense. The SmartSantander project uses vehicles as part of a city-wide and multi-purpose wireless sensor network [19]. It employs a three-tier architecture, with a server tier, a gateway tier, and an IoT node tier. A testbed was developed, involving applications such as parking management and parks’ irrigation. The work by Alsina-Pagés et al. designs and evaluates a bus-based sensor network to monitor noise in a smart city [20]. The work evaluates costs and necessary equipment to implement such a service.

Opensense [21] and Mosaic [22] use vehicles to monitor the air quality in the urban environment, proposing models and algorithms to improve the accuracy of the measurements obtained by the system. The Opensense project also embeds sensors into urban vehicles, using models to estimate pollution in areas that are not reached by sensors. In Mosaic, sensors are embedded into urban vehicles and send data to a cloud service, using a GSM/GPRS module. In the cloud, Artificial Neural Networks (ANN) and Support Vector Machines (SVM) are used to remove inaccurate data. The cloud also estimates pollution in areas not reached by the sensors. In a certain sense, these works improve the coverage of the system, since they estimate pollution in areas not reached by sensors. This procedure is possible for pollution, but probably unfeasible for some applications, such as pothole detection. One more important remark is that another work from the Mosaic team proposes a coverage model dividing areas into grids, with different weights for tiles containing some point of interest [23]. According to their results, the model works adequately for pollution application. Nevertheless, the model is not designed for applications that need a street-wise notion of coverage, such as pothole detection or traffic monitoring.

Some works use Google Street View cars to gather data about a city environment, taking advantage of the fact that these cars have predictable trajectories. The work by Apte et al. uses Google Street View cars to provide measurements on air quality in the city of Oakland [24]. The work also proposes models to apply the same methods to different cities. Also using Google Street View cars, the work by Von Fischer et al. uses sensors to rapidly detect gas leaks and their sources.

The work of Fiore et al., differently from other related work, explores signal processing models to estimate the accuracy of measurements obtained by vehicles and pedestrians [25]. Their work considers that every vehicle is equipped with sensing nodes and uses the density of devices in a city to define the sensing quality in a given region.

The works of Zhu et al. and Du et al. both analyze the effectiveness of vehicular networks to monitor urban traffic. Du et al. propose models to use sensors available in cars to estimate traffic speed and intensity [26]. Zhu et al. study the coverage and the quality of data generated by a taxi fleet of approximately 4000 vehicles in the city of Shanghai [27]. The present work performs an analysis of a different number of sensing nodes on a similar network. The studied network though is not limited to the monitoring of urban traffic and employs only urban buses in the gathering of data.

Zhao et al. study the opportunistic coverage of a city using different mobility models [16]. Zhao et al. characterize coverage of sub-regions by the probability of visit by a vehicle equipped with a sensing node [16]. Our work models a network composed of vehicles with deterministic routes, in contrast with a stochastic model.

The previous work by Ali and Dyo considers the coverage of a bus-based sensor network for road surface inspection [9]. Their work presents a coverage model based on the concept of a street segment, defined as a part of a street between two adjacent intersections. The authors then formulate a MILP based on the maximum coverage problem and propose a greedy algorithm to approximate a solution. The method is tested on a dataset of London’s bus routes, by applying the coordinates of the routes to a turn-by-turn API and using an additional method to identify the segments. In our work, we base our coverage model on using a slightly different definition of street section, which is not necessarily between two adjacent intersections and has a length associated with it. Additionally, we propose a different MILP formulation that takes street section lengths into account. Finally, we use the road id offered by Google Snap to Roads to identify every street section.

In a previous work, we proposed SensingBus, a system where sensing nodes are coupled into buses and gateways are placed in the city to implement opportunistic data delivery [5]. In the context of SensingBus, we discuss the data delivery delay of a bus-based sensor network [17]. The data delivery delay depends on the amount and the positioning of gateways in the network. The present work analyzes the spatial coverage of the SensingBus network. Both the delivery delay and the spatial coverage are important metrics to assess the viability and cost-effectiveness of a mobile sensor network based on urban transportation vehicles.

## 6. Conclusions and Future Work

Covering an entire city with sensing (and communicating) equipment can be extremely expensive. An option to reduce costs and still obtain significant spatial coverage is to use mobile sensing nodes. This approach increases the area sensed by each node while enabling opportunistic communication with gateways, which avoids the cost of an infrastructure network covering the entire city. An affordable way of moving sensors around the city is to embed them into urban buses.

In this work, we have analyzed the spatial coverage of a mobile sensor network based on urban buses. A coverage model was proposed as a function of the street sections to be sensed, the set of buses equipped with sensing nodes and their paths in the city. A mixed-integer linear programming problem was formulated to maximize the coverage when there is a budget limiting the number of buses that are equipped with sensing nodes. We applied the problem to real data produced from the buses of the city of Rio de Janeiro. These data were gathered as raw GPS coordinates from buses and pre-processed using Google Snap to Roads to transform the coordinates into street sections. In the last pre-processing step, we estimated the length of each street section and validated it using information from other datasets. After that, we used these data as input to the formulated MILP problem. The results obtained showed that with only 18% of the fleet of Rio de Janeiro, we can cover at least 94% of the total area, which would be covered by the whole fleet. Interestingly enough, we show that only 32 buses can cover at least 40% of the total covered by all buses. This indicates that it is possible to establish an initial service at very low cost and incrementally deploy a system that serves the whole city. Finally, the results also show the relationship between the number of buses, spatial coverage and the number of times that a specific street section is visited during the day.

As future work, we aim to explore the information on bus lines and their paths to maximize the spatial coverage. Assuming that buses serving the same line follow the same path, this strategy can lower the number of variables of the optimization problem. One challenge is to determine and prove that this assumption is true in practice or propose some measurement of the path deviation. We will also investigate strategies to solve the problem more efficiently and deepen our analysis of time coverage, proposing a coverage model that also takes into account the interval between visits a street section gets. Another aspect to investigate in SensingBus is the different data delivery strategies that can be employed, other than V2I. For instance, private vehicles could receive data in a vehicle-to-vehicle (V2V) fashion and forward them to the nearest bus stop.

## Figures and Tables

**Table 1:** Notations used in this work.

Notation	Description	Type
I	Street sections that can be covered	Set
$I_{s}$	Street sections covered by the buses chosen in the problem output	Set
J	Urban buses	Set
$J_{s}$	Buses equipped with sensing nodes, chosen in the problem output	Set
$N_{i}$	Buses that can cover street section *i*	Set
$T_{j}$	Street sections that can be covered by bus *j*	Set
$a_{i}$	Value indicating the length of the street section *i*	Parameter
*p*	Total number of buses to be equipped with sensing nodes	Parameter
$x_{j}$	Binary value indicating if bus *j* is chosen to be equipped with a sensing node	Variable
$y_{i}$	Binary value indicating if street section *i* is covered	Variable
*C*	Total coverage of the city	Variable

**Table 2:** Attributes of the gathered and estimated datasets.

Attribute	Value	Dataset
Total gathered positions (#)	5,496,878	Gathered
Total buses in original set (#)	6075	Gathered
Removed positions after filtering (#)	1,384,925	Gathered
Total positions after filtering (#)	4,111,953	Gathered
Removed buses after filtering (#)	328	Gathered
Total buses after filtering (#)	5747	Gathered
Total positions after estimation (#)	52,250,671	Estimated
Total street sections (#)	95,992	Estimated
Sum of all street section lengths (km)	5655	Estimated
Total distance traveled (km)	1,005,327	Estimated
